# Biopsychosocial determinant of quality of life of older adults in Pakistan and Canada

**DOI:** 10.3389/fpsyt.2024.1364443

**Published:** 2024-03-11

**Authors:** Syeda Shahida Batool, Samra Tanveer, Sarvjeet Kaur Chatrath, Syeda Azra Batool

**Affiliations:** ^1^ Department of Psychology, Government College University, Lahore, Pakistan; ^2^ Canberra Business School, Canberra University, Canberra, ACT, Australia; ^3^ School of Economics, Bahauddin Zakariya University, Multan, Pakistan

**Keywords:** quality of life, health and lifestyle, self-efficacy, self-esteem, social support

## Abstract

**Background:**

The rapidly rising average age of the older adults has brought various global healthcare challenges. A core challenge is how to enhance their quality of life (QoL).

**Objective:**

The objective of the current study was to test the significance of biopsychosocial determinants of quality of life of older adults in Pakistan and Canada.

**Methodology:**

A cross-sectional survey was carried out on a conveniently approached purposive sample of 1,005 older adults (Pakistani = 557 and Canadian = 448) of age range between 60 years and 80 years. The data were collected via demographic datasheet, World Health Organization Quality of Life Brief Scale, Health and Lifestyle Questionnaire, General Self-Efficacy Scale, Rosenberg Self-Esteem Scale, and Berlin Social Support Scale.

**Results:**

The results of hierarchical regression analysis showed that biopsychosocial factors (*viz.*, health and lifestyle, chronic illness, self-efficacy, self-esteem, and social support) significantly predicted (*R* ^2^ = .27, and.68) quality of life of older adults in Pakistan and Canada, respectively, after controlling the demographic variables. Significant differences were found between Pakistani and Canadian older adults on biopsychosocial factors: Canadian older adults scored significantly higher on health and lifestyle, self-efficacy, and quality of life, and older adults in Pakistan scored significantly higher on self-esteem and social support.

**Conclusion:**

A significant amount of better QoL of older adults can be achieved through enhancing the biopsychosocial correlates of their QoL, both in Pakistan and Canada.

## Introduction

The average age of the older adults is rising rapidly (1), and this is a big global challenge. Most developed and developing regions of the world are facing much rapid increase in older individuals of 65 years and above. By 2040, the world is projected to have 1.3 billion older adults who will account for 14% of the global population (2). This extraordinary upsurge in aging has brought numerous changes in the world, for example, exploration of risk and protective factors of their well-being, need for professionals who are specialized in geriatric care, and need for effective legislation focused on addressing the needs of this rapidly increasing aging population.

Like many social behaviors, aging is a culturally knitted phenomenon. Individuals living in different parts of the world pursue their own internalized cultural values with age (3). Personal characteristics, social relationships and interactions, cognition, and health status of older adults may not be manifested in exactly the same way across cultures. Cultural differences in health-related behaviors, psychological beliefs, and social participation have been proposed as possible mechanisms explaining health patterns of aging population (e.g., 4, 5). Empirical studies have been undertaken to compare health across nations/countries to assess how differences in macro-social and economic contexts shape individual health and well-being (e.g., 6, 7).

Like other parts of the world, a rapid increase in the aging population is a major challenge for both Pakistan and Canada. According to 2017 census, the individuals 65 years and above account for 4.48% of the total population in Pakistan (8). The number of individuals above 60 years is going to increase to 23.76 million in 2030 in Pakistan (9). Similarly, the older Canadians accounted for 12% in 2010 and will account for 23% in 2030 (10). Pakistan and Canada are two countries, diverse in terms of culture, and socioeconomic growth and development. Public policies and healthcare system in Pakistan are not conducive to the well-being of older adults. Older adults in general do not receive any financial support from the government. Legislation for elderly population is in its embryonic stage. The first major Senior Citizens Bill (2019) focuses on safeguarding fundamental rights of citizens (11). On the other hand, the government of Canada is presently focusing on the provision of a wide range of benefits to the older adults (12). Older adults are classified as a high-risk population in terms of health and quality of life (QoL). A core challenge due to this rapidly increasing high-risk aging population across Pakistan and Canada is to consider how to enhance their QoL. To achieve this goal, the United Nations has developed a research agenda for the twenty-first century to gain insights about the determinants of good QoL or healthy aging (13). The agenda focuses on the identification of interventions, curative measures, and rehabilitative models that can be used for gaining a better understanding about the processes and dynamics of aging in addition to the determinant factors and their roles (14). In terms of healthy aging, the constituents of a good life and correlates of a better QoL have remained crucial. Findings indicate that older adults in the developed regions (e.g., Canada) remain in good health for longer periods of time. A more detailed insight can be gained only through the assessment of cross-cultural differences in quality of life in terms of its biopsychosocial correlates. In the present study, the World Health Organization’s (WHO) definition of quality of life as an individual’s perception of their position in life in the context of the culture and value systems in which they live and in relation to their goals, expectations, standards, and concerns has been followed.

### Health, lifestyle and QoL of older adults

Individuals’ lifestyle and behaviors influence their health and quality of life (15). Good quality of life has been found to motivate people to make lifestyle changes. Consumption of healthy food is associated with a good quality of life (16). Biological conditions and lifestyle (e.g., chronic illness, healthy diet, mobility, and exercise) play significant roles in the QoL of older adults. Older adults who live with chronic conditions experience declines in their health-related quality of life (17). However, encouraging physical activities (exercise), a balanced diet, and enhancements in the biological aspect of the functioning of older adults can reduce their disability, which can lead to a substantial impact in fostering improvements in QoL (5, 11). Healthy diet including berries and other fruits has been found to be beneficial in improving the flow of antioxidant and the insufficiencies and plays role in the improvements in chronic conditions (for detail, see 18).

### Self-efficacy, self-esteem, and QoL of older adults

Empirical literature indicates that the presence of certain psychological attributes have a significant impact on the well-being of older adults (19).

Self-efficacy is an important attribute, marked by the ability of individuals to produce desired outcomes or the amount of confidence that they attach to themselves in achieving specific desirable outcomes through their behaviors or belief systems (20). Belief in their efforts and success determines psychological well-being, life satisfaction, health outcomes, and overall QoL of older adults (21). Studies have shown significant positive relationship between self-efficacy and quality of life (22).

Self-esteem is another psychological construct defined as a global sense of self-worth or the amount of value an individual attaches to himself or herself (23). High self-esteem is associated with pleasant feelings of happiness, but it does not mean that raising self-esteem alone is going to lead to a considerable amount of impact on the psychological well-being of individuals (24). Self-esteem has been proven to play central role in improving the quality, and empirical studies have shown significant positive relationship between self-esteem and various dimension of quality of life (25). Chronic diseases in older population are a major public health concern, and older adults who had high self-esteem scored higher on quality of life (26).

### Social support and QoL of older adults

Social support is referred to as the interpersonal processes and supportive mechanisms that are used for maintenance as well for the promotion of health and well-being (27). Sun et al. (24) found that older adults rely extensively on their sources of social support including friends, family, relatives, and colleagues. Family and other sources of social support help older adults in meaning making and ensure them that they are important and being valued and promote their resilience. Social engagement and social support minimize loneliness and positively influence quality of life in old age and also bring a reduction in depressive symptoms (27).

### Cross cultural differences in QoL of older adults

Empirical studies indicate that culture determines the number of differences in QoL of older adults. Individualistic cultures emphasize independence, psychological attributes, and governmental backing and support for fulfillment of health-related needs of the older adults. Social determinants of QoL differ in various cultures (28). Family is an important unit of social support in collectivist cultures that support to investigate the social aspects in the determination of QoL of the older adults in comparison to those living in an individualistic culture (29).

Studies have also concluded that older adults in Pakistan, who have high social support of family friends and the community, had access to healthcare system and were engaged in social services and social participation, reported better QoL than those who were living separately and perceived lesser social support (30–32). Similarly, Plouffe (33) found that older adults in Canada, who scored higher on loss of economic independence, lack of access to healthcare services for treatment of chronic conditions, and loss of social support, experienced low QoL (34). Studies have found that socioeconomic conditions and access to healthcare services (35) are significant predictors of the health outcomes and QoL.

In a nutshell, access to healthcare facilities, better physical health status, higher self-efficacy and self-esteem, and provision of social support, positively predict better QoL of older adults living in various parts of the world. Studies have also shown cultural differences in terms of the role of social support, living conditions, and personal/psychological attributes of older adults in their QoL (36). Moreover, a socioeconomic and political analysis of Pakistan and Canada indicates that older adults in Canada receive higher levels of governmental support and backing from formal community programs and receive quality healthcare services. The lack of such supportive structures for the older adults in Pakistan inspired to carry out the current cross-cultural study on older adults living in these two countries of diverse cultures and healthcare systems.

### Rationale of the study

Older individuals are the most vulnerable segment of population to the health-related QoL; thus, healthy aging is a core healthcare concern of WHO. Developed countries and a wide range of healthcare entities are collaborating on providing the best socioeconomic, psychological, and physical conditions that can contribute towards healthy aging and better QoL of the older adults. Unless we unleash the factors that play substantial role in a better QoL of older adults via empirical studies, we cannot attain this goal. Literature indicates the significance of various biopsychosocial and demographic variables in QoL of older adults, but much work has been done in the west and little cross-cultural research exists that assessed the predictive strength of these variables in the countries of diverse cultures in the context of QoL of older adults. To address this literature gap, and keeping in view the diversity of intricating social system and healthcare facilities in Pakistan and Canada, we carried out this study to investigate relative strength of biopsychosocial predictors of QoL of older adults in Pakistan and Canada.

### Hypotheses

H_1_: Biopsychosocial variables (health and lifestyle, self-efficacy, self-esteem, and social support) positively predict the quality of life of older adults in Pakistan and Canada, after controlling demographic variables;

H_2_: Older adults in Pakistan significantly differ on quality of life and biopsychosocial variables from the older adults in Canada.

## Materials and methods

### Sample

The sample of the study was selected from Pakistan and Canada via a purposive sampling technique. The data were collected from 1,005 (men= 620 and women= 385) older adults (Pakistani=557 and Canadian= 448) of age range between 60 and 80 years (*M*= 68.70, *SD*= 5.65). Individuals who reported history of psychiatric conditions, fatal disease, and disability, and were living in nursing homes were not included in the study. Moreover, sample characteristics were also comparable in terms of age range and levels of education (high school to university graduates). Minimum criteria of sample size for regression analysis and structural equation modeling determined the sample size.

### Measures

To qualify the assessment of study variables in Pakistan and to control the language barrier, all scales were translated from English into Urdu. A standardized procedure of translation, guided by Brislin (13), was followed. For cross-language validation and establishment of psychometrics of both Urdu and English versions of all the scales, confirmatory factor analysis and reliability analysis were run, and all scales showed promising validity and reliability in both languages on the samples of Pakistan and Canada separately (37).

#### Demographic datasheet

It covered demographic variables that showed the basic characteristics of the sample (e.g., age, gender, education, and marital status).

#### World Health Organization Quality of Life

We used the World Health Organization Quality of Life (WHOQoL-BREF) (38) consisting of 26 questions to measure the quality of life of older adults in the current study. One question covers general QoL; the other one is about the level of satisfaction with health and the remaining questions deal with the four core domains of QoL including physical (e.g., Do you have enough energy for everyday life)?, psychological (e.g., To what extent do you feel your life to be meaningful)?, social (e.g., How satisfied are you with the support you get from your friends)?, and environmental (e.g., How safe do you feel in your daily life)?. The response range is from 1 to 5. Domain scores are scaled in a positive direction (i.e., higher scores denote higher quality of life). Internal consistency ranged from.60 to.88 and.53 to.88 for Urdu and English versions respectively in the current study.

#### Health and lifestyle questionnaire

A questionnaire to measure the biological component of the biopsychosocial model was used, and items of the scale were constructed by following Tartaglia (39) and with the consultation of experts. It comprises seven items, and the response format is a 5-point Likert scale (1 = not at all, 2 = a little, 3 = moderately, 4 = mostly, and 5 = completely). All items of the questionnaire are positively phrased, and participants’ responses are added to obtain a total score. Higher score indicates better status of health and lifestyle. The reliability of the scale was.72 and.78, respectively, for Pakistan and Canada in the current study.

#### General self-efficacy scale

The psychological component of the biopsychosocial model was measured via General Self-Efficacy Scale (40). This scale comprises 10 items, and the response format is a 4-point Likert scale (1 = not at all true, 2 = hardly true, 3 = moderately true, 4 = exactly true). A higher score indicates greater self-efficacy. The scale showed Cronbach’s alpha values= .89 and.80, respectively, for Pakistan and Canada in the current study.

#### Rosenberg self-esteem scale

The psychological component of the biopsychosocial model was measured via Rosenberg Self-Esteem Scale (41). The scale comprises 10 items, and the response format is a 4 point Likert scale (1 = strongly disagree, 2 = disagree, 3 = agree, and 4 = strongly agree). Items number 2, 5, 6, 8, and 9 were reverse. A higher score indicates higher self-esteem. Alpha reliabilities of the original English version ranged from.72 to.87. It has high criterion validity (41). The scale showed Cronbach’s alpha values= .72 and.69, and mean scores on the subscale were =29.10 and 28.31, respectively, for Pakistan and Canada in the current study.

#### Berlin social support scale

The social component of the biopsychosocial model was measured via Berlin Social Support Scale (17). Items are scored on a 4-point Likert scale. Possible endorsements are strongly disagreeing, (1) somewhat disagree, (2) somewhat agree, (3) and strongly agree (4). For the present study, Perceived Social Support (items=8) subscale was used for data collection. The scale showed Cronbach’s alpha ranging between.84 and.78, respectively, for Pakistan and Canada in the current study.

### Procedure

After the approval of the research proposal from Advanced Studies and Research Board of Government College University Lahore, Pakistan, participants of the study were contacted, and data collection was initiated in January 2020 and completed in December 2020. Participants were verbally briefed about the purpose and nature of the study. They were contacted at their homes, shopping malls, community centers, and at healthcare centers. The second author personally contacted the participants and herself collected the set of questionnaires immediately after the participants completed their responses. It took a total of 12 months to complete data collection from Pakistan and Canada. Each participant took 30–45 min to complete all questionnaires.

Ethical considerations were fully observed. Before data collection, permission was sought from authors of the scales. Informed consent paper was signed by all participants. Confidentiality and anonymity were ensured. Moreover, the participants were informed about their right to withdraw. Certain domains of ethics in geriatric research were followed (e.g., legal competence and mental stability) of the participants.

### Analysis

Two hierarchical regression analyses were carried out to assess the significant predictive strength of biopsychosocial factors in the quality of life of older adults in Pakistan and Canada after controlling demographic variables.

## Results

Initially, zero-order correlations were calculated among demographic variables, biopsychosocial factors, and QoL of older adults, separately on Pakistani and Canadian samples (see *Annexure A*). The results showed that all biopsychosocial variables and most of demographic variables had significant inter-correlations, which supported to run further statistical analyses.

Independent variables/predictors given in **Tables 1** and **2** are health and lifestyle, self-efficacy, self-esteem, and social support, and dependent/outcome variables are quality of life total, psychological, and environmental features. Demographic variables are used as controlled variables in the analyses.

**Table 1:** Demographics and biopsychosocial variables as predictors of quality of life as outcome (Pakistan, n = 557).

Predictors	*ΔR* ^2^	B
Step 1	.13	
Gender		.14**
Income		.03
Children with ASD		.11**
Living place		.15**
Family system		−.07
Step 2	.27	
Self-esteem		.30**
Chronic illness		.22**
Social support		.17*
Health and lifestyle		.16**
Self-efficacy		.10*
Total Δ*R* ^2^	.40	

**p <.01, *p <.05.

**Table 2:** Demographics and biopsychosocial variables as predictors of quality of life as outcome (Canada, n = 448).

Predictors	*ΔR* ^2^	B
Step 1	.07	
Gender		.04
Income		.21**
Children with ASD		.05
Living place		.01
Family system		.11*
Step 2	.61	
Social support		.44**
Self-efficacy		.24**
Health and lifestyle		.16**
Self-esteem		.15**
Chronic illness		.15**
Total Δ*R* ^2^	.68	

**p<.01, *p<.05.

**Table 1** shows that models 1 and 2 are significant, i.e., *F_1_* (7, 521) = 11.52, *p* <.001 and *F_2_* (11, 517) = 31.97, *p* <.001. For model 1, Δ*R* ^2^ is 13%; for model 2, Δ*R* ^2^ is 27%. Findings indicate that self-esteem (β = .30, *p*<.01), chronic illness (β = .22, *p* <.01), social support (β = .17, *p* <.01), health and lifestyle (β = .16, *p* <.01), and self-efficacy (β = .10, *p* <.05) significantly predicted 27% of variance in QoL of older adults in Pakistan, after controlling the impact of demographic variables.

**Table 2** shows that models 1 and 2 are significant, i.e., *F_1_* (7, 432) = 4.94, *p*<.001 and *F_1_* (11, 428) = 83.65, *p* <.001. For model 1, Δ*R* ^2^ was 7%; for model 2, it is 61%. Findings suggest that social support (β = .44, *p*<.01), self-efficacy (β = .24, *p*<.01), health and lifestyle (β = .16, *p*<.01), self-esteem (β = .15, *p* <.01), and chronic illness (β = .15, *p* <.01) significantly predict 61% variance in QoL of older adults in Canada by controlling the demographics.

In order to compare the group differences, independent sample t-test of was run.

**Table 3** indicates that there are significant mean differences between older adults in Pakistan and Canada on QoL (*t* = −4.44, *p ≤*.001). Canadians scored higher on quality of life as compared to Pakistani older adults. Significant mean differences also appear between the groups with respect to health and lifestyle (*t* = −6.72, *p* ≤.000), self-efficacy (*t* = −2.11, *p* ≤.035), self-esteem (*t* = 2.46, *p ≤*.014), and social support (*t* = 4.86, *p* ≤.001). It is found that Canadian older adults have higher score on health and lifestyle and self-efficacy, whereas Pakistani older adults have higher scores on self-esteem and perceived social support. The Cohen’s *d* indicates low to moderate effect size of independent variable on dependent variable (ranges from.13 to.42). The values of Cohen’s *d* reflect that health and lifestyle, social support, and quality of life have more weight in one country than in another.

**Table 3:** Mean differences on biopsychosocial factors and quality of life of elderly in Pakistan and Canada (N =1,005).

Variable	Pakistan (*n* = 557)		Canada (*n* = 448)				95% *CI*		
	*M*	*SD*	*M*	*SD*	*t (1003*)	*P*	*LL*	*UL*	Cohen’s *d*
Health and lifestyle	20.73	4.80	22.70	4.39	−6.72	.000	−2.55	−1.39	.42
Self-efficacy	27.46	6.40	28.29	5.73	−2.11	.035	−1.58	−.05	.13
Self-esteem	29.10	4.89	28.31	5.22	2.46	.014	.16	1.41	.15
Social support	24.94	4.46	23.50	4.91	4.86	.000	.86	2.02	.30
Quality of life	80.09	13.48	84.09	15.01	−4.44	.000	−5.76	−2.23	.28

CI, confidence interval; LL, lower limit; UL, upper limit; N = 1,005.

## Discussion

The study was carried out to assess predictive strength of biopsychosocial factors to predict quality of life of older adults in Pakistan and Canada. Hierarchical regression analyses were run to test the hypotheses based on the objectives of the study. Demographic variables were treated as controlled variables in the regression analyses, as zero-order correlation in the current study and the literature direct that demographic variables have a significant relationship with QoL of older adults (42). The results showed that chronic illness and health and lifestyle (biological factors) significantly predicted quality of life of the participants in both countries (see **Tables 1**, **2**). The findings of the present study are in line with the earlier studies, supporting the claim that poor lifestyle and presence of chronic diseases have a negative impact on QoL (e.g., 43). Literature indicates that experiencing biological health problems has a causal influence on QoL, self-esteem, and self-efficacy whereas satisfaction with biological aspects of health is one of the most significant predictors of QoL and emotional functioning among the older adults (44). Active lifestyle and taking part in social activities maximize physical abilities of older adults; they are able to structure their day-to-day routine, and their engagements with these activities help them in the replacement of their losses and is helpful to boost their QoL and well-being (45). Unhealthy lifestyle increases the probability of chronic illnesses in old age, which consequently impairs their perceptions about QoL. There is empirical evidence that older adults are primarily concerned with the physical aspects of their health, that is why healthy lifestyle and satisfaction with health have significant impact on their quality of life (44). The findings of the study have suggested that optimal level of physical health and consumption of healthy food, mobility, and regular exercise ensure better health and QoL of older individuals.

Self-efficacy and self-esteem (psychological factors) also appeared as significant predictors of QoL of older adults in Pakistan and Canada (see **Tables 1**, **2**). Results are in line with a number of studies that highlighted the importance of self-efficacy in relation to QoL of older adults (46). Self-efficacy embarks the belief and confidence in older adults that they can perform their day-to-day tasks without relying on others because it allows them to explore and exert control over their immediate environment (43), accomplish their life goals (47), and the excellence with which they are able to perform tasks related to their daily living (45). Self-efficacy has a great deal of relevance to adherence with different forms of healthcare guidelines (48).

Self-esteem as a significant positive predictor of QoL in older adults in the current study is consistent with the findings of previous studies (49) that support the auxiliary role self-esteem in the mental and physical health of older adults and the level of independence that they exhibit on a daily basis, social relations, and environmental domain of QoL. Promoting self-esteem of the older adults has positive impact on their motivation, encourage them to face the adversities of old age, and instill in them confidence and sense of control over their life (46). Self-esteem and self-efficacy encourage individuals who have even restricted residual physical abilities to participate in social activities (50) that will give a higher meaning and purpose to their life, which in turn can positively contribute towards their QoL.

Social support as a positive predictor of QoL of older adults in both countries is in accordance with the studies that have concluded that quality of life among the older adults is determined by the availability and non-availability of social support services to them, marked by personal relations and the kind of support available to them from friends and family (51). Social support not only serves as a source of emotional and physical support but is also a basis of promoting active engagement with the society and environment.

Although predictors of QoL in both countries are similar, the older adults in Canada scored significantly higher on health and lifestyle, self-efficacy, and overall quality of life, and the older adults in Pakistan scored higher on self-esteem and social support (see **Table 3**). Different contextual factors might be attributed to the higher scores of Canadian older adults on these variables. Canadian older adults are more aware of health-related behaviors, more knowledgeable about their health-related quality of life, have better access to healthcare services, income security, social networking, and excellent transportation (34, 52, 53). The Canadian economic and social systems provide more rights to the older adults in comparison to the Pakistani systems of governance. It is pertinent to note that the higher score of Canadian older adults on self-efficacy and quality of life in comparison to older adults in Pakistan validates the previous studies that reported higher scores of older adults in Canada on these variables in comparison to a number of other countries, for example, China, Japan, Brazil, USA, France, Germany, and India (54). We may conclude that better access to legal support and healthcare facilities and social support interventions inculcates high self-efficacy in Canadian older adults and supplements their healthy psychosocial and physical functioning.

Higher scores of older adults in Pakistan on self-esteem and social support could be attributed to close knitted social structure of Pakistan. Being a collectivistic society, the availability of social support from children, friends, and family for the older adults boosts their self-esteem (55). In Pakistan, children are expected to take care of their older parents and to fulfill their physical, psychological, social, and emotional needs. It is also marked for being a core cultural and religious obligation, which might account for the explanation of the higher scores of the Pakistani older adults on these factors. Pakistan is marked for being a collectivist society (56), in comparison to Canada, which is a highly individualistic society (57). This can possibly contribute towards the Canadian older adults, relying on the government and themselves for their socioeconomic and healthcare needs in comparison to Pakistani older adults, who mainly rely for support on their children and families. Results are consistent with (11, 58) that older adults in Pakistani generally score high on social support and self-esteem and family plays positive role in providing social support that has positive impact on the well-being of Pakistani older adults. Results are also in line with (59) that in collectivistic cultures, older adults have greater availability of social support to perform activities of daily living, and they have higher self-esteem.

### Implications

Findings of the study recommend to provide better healthcare services, opportunities for physical engagement, old age friendly gyms, financial and legal support, and healthcare coverage to the older adults to ensure their better QoL. The older adults should be motivated to adopt active and healthy lifestyle. The study has implications for families to play a major part in providing psychosocial and emotional support and build connections to improve self-worth, self-esteem, and self-efficacy of older adults that lead to better QoL. Media can play effective role in providing informational support and mass public awareness on the protective and risk factors of QoL of older adults.

The findings emphasize the need of interventions that can boost self-efficacy of the older adults that will possibly impact their overall quality of life and will have positive impact on a number of dimensions of their psychosocial and emotional functioning.

Gerontology should be taught as a compulsory subject at higher education institutions that may cover needs and health-related issues of older adults to improve their QoL. Results have implications for the government of Pakistan to introduce all-inclusive policies and legislative measures and improve healthcare facilities that can bring improvements in the QoL of the older adults. The government of Canada is recommended to revisit their social fabrication to encourage and incentivize younger generation to provide emotional support to older adults that will boost their self-esteem. The focus of all policies should be to create an older adult’s friendly environment.

### Limitations and future suggestions

The study is correlational in nature, so the presence of causality cannot be assumed. In order to study the causal model, experimental and longitudinal studies are recommended. All of the measures used in the study were self-reported measures, so we cannot ignore the likely common method variance in the results. We assessed only protective factors (positive attributes as predictors of QoL); in future studies, risk factors (e.g., loneliness, ostracism, pessimism, and loss of meaning) should also be studied.

## Conclusion

On the basis of the results, we conclude that a significant amount of better QoL of older adults can be achieved through enhancing the biopsychosocial correlates of their QoL, both in Pakistan and Canada. Relatively higher scores of older adults in Pakistan on social support and self-esteem support the notion that the provision of social support promotes self-esteem of older adults that ensures their QoL. In countries like Pakistan, which is undergoing rapid social changes and where older adults do not have considerable awareness of their health and related factors and have limited access to government-assisted health facilities, social factors reflecting engagement of significant others, especially offspring and grandchildren, appeared to be more important in the better QoL of older adults. On the other hand, comparatively higher scores of the older adults in Canada on healthy lifestyle and behavior, self-efficacy, and QoL make it evident that older adults in Canada are more health conscious and have high-quality healthcare system. It seems that old-people-friendly legislation in Canada has inculcated self-confidence in them for the self-management of their health-related challenges, and this all has a considerable influence on their better QoL.

## Data availability statement

The original contributions presented in the study are included in the article/**Supplementary Material**. Further inquiries can be directed to the corresponding author.

## Ethics statement

The studies involving humans were approved by Advanced Studies and Research Board of Government College University Lahore. The studies were conducted in accordance with the local legislation and institutional requirements. The participants provided their written informed consent to participate in this study.

## Author contributions

SSB: Conceptualization, Data curation, Formal analysis, Methodology, Software, Supervision, Visualization, Writing – original draft, Writing – review & editing. ST: Conceptualization, Data curation, Investigation, Resources, Writing – review & editing. SC: Data curation, Validation, Visualization, Writing – review & editing. SAB: Methodology, Validation, Visualization, Writing – review & editing.
