# Supplementary material for: Biopsychosocial determinant of quality of life of older adults in Pakistan and Canada
Source: Front Psychiatry. 2024 Mar 11;15:1364443. doi: 10.3389/fpsyt.2024.1364443 (PMC10961405; doi:10.3389/fpsyt.2024.1364443)
Supplement: Supplementary file 1 [file Table_1.docx]

| **Annexure-A**  Table 3 | | | | | | | | | |
| --- | --- | --- | --- | --- | --- | --- | --- | --- | --- |
| *Inter-correlations among Biopsychosocial Variables and Quality of Life of Elderly in Pakistan* (*n* = 557) | | | | | | | | | |
| Variable | 1 | 2 | 3 | 4 | 5 | 6 | 7 | 8 | 9 |
| 1.Lifestyle | __ | .38 | .30 | .15 | .37 | .30 | .30 | .18 | .35 |
| 2.Self-Eff. |  | __ | .43 | .42 | .42 | .36 | .44 | .22 | .31 |
| 3.Self-Est. |  |  | __ | .42 | .49 | .43 | .47 | .23 | .37 |
| 4.SS |  |  |  | __ | .38 | .28 | .41 | .24 | .27 |
| 5.WHOQOL |  |  |  |  | __ | .83 | .80 | .64 | .84 |
| 6.Physical |  |  |  |  |  | __ | .56 | .41 | .52 |
| 7.Psych |  |  |  |  |  |  | __ | .43 | .56 |
| 8.Social |  |  |  |  |  |  |  | __ | .45 |
| 9.EF |  |  |  |  |  |  |  |  | __ |
| *Note*. Lifestyle = health and lifestyle, Self-Eff. = self-efficacy, Self-Est. = self-esteem, SS = social support, WHOQOL = quality of life total, Psych = psychological, EF = environmental features.  All correlation coefficients significant at *p* < .001 (two-tailed) | | | | | | | | | |

Table 1 shows that health and life style scores significantly [low to moderately] correlates with self-efficacy (*r* = .38, *p*<.001), self-esteem (*r* = .30, *p*<.001), social support (*r* = .15, *p*<.001), quality of life (*r* = .37, *p*<.001), physical health (*r* = .30, *p*<.001), psychological condition (*r* = .30, *p*<.001), social (*r* = .18, *p*<.001), environmental domain (*r* = .35, *p*<.001).

Self-efficacy significantly [moderately] correlates with self-esteem (*r* = .43, *p*<.001), social support (*r* = .42, *p*<.001), quality of life (*r* = .42, *p*<.001), physical health (*r* = .36, *p*<.001), psychological condition (*r* = .44, *p*<.001), social (*r* = .22, *p*<.001), environmental domain (*r* = .31, *p*<.001).

Self-esteem significantly [moderately] correlates with social support (*r* = .42, *p*<.001), quality of life (*r* = .49, *p*<.001), physical health (*r* = .43, *p*<.001), psychological condition (*r* = .47, *p*<.001), social (*r* = .23, *p*<.001), environmental domain (*r* =.37, *p*<.001).

Social support significantly [moderately] correlates with quality of life (*r* = .38, *p*<.001), physical health (*r* = .28, *p*<.001), psychological condition (*r* = .41, *p*<.001), social (*r* = .24, *p*<.001), environmental domain (*r* =.27, *p*<.001).

| **Table 2** | | | | | | | | | |
| --- | --- | --- | --- | --- | --- | --- | --- | --- | --- |
| *Inter-correlations among Biopsychosocial Variables and Quality of Life of Elderly in Canada* (*n* = 448) | | | | | | | | | |
| Variable | 1 | 2 | 3 | 4 | 5 | 6 | 7 | 8 | 9 |
| 1.Lifestyle | __ | .52 | .48 | .45 | .57 | .50 | .45 | .36 | .51 |
| 2.Self-Eff. |  | __ | .57 | .51 | .63 | .53 | .54 | .37 | .56 |
| 3.Self-Est |  |  | __ | .63 | .64 | .51 | .56 | .40 | .59 |
| 4.SS |  |  |  | __ | .74 | .58 | .56 | .55 | .69 |
| 5. QOL |  |  |  |  | __ | .84 | .79 | .66 | .90 |
| 6.Physical |  |  |  |  |  | __ | .55 | .47 | .65 |
| 7.Psych |  |  |  |  |  |  | __ | .40 | .64 |
| 8.Social |  |  |  |  |  |  |  | __ | .52 |
| 9.EF |  |  |  |  |  |  |  |  | __ |
| *Note*. Lifestyle = health and lifestyle, Self-Eff. = self-efficacy, Self-Est.= self-esteem, SS = social support, QOL = quality of life total, Psych = psychological, EF = environmental features.  All correlation coefficients significant at *p*< .001(two-tailed) | | | | | | | | | |

Table 2 shows that health and lifestyle scores significantly [moderately] correlates with self-efficacy (*r* = .52, *p*<.001), self-esteem (*r* = .48, *p*<.001), social support (*r* = .45, *p*<.001), quality of life (*r* = .57, *p*<.001), physical health (*r* = .50, *p*<.001), psychological condition (*r* = .45, *p*<.001), social (*r* = .36, *p*<.001), and environmental domain (*r* = .51, *p* < .001).

Self-efficacy significantly [moderately] correlates with self-esteem (*r* = .57, *p*<.001), social support (*r* = .51, *p*<.001), quality of life (*r* = .63, *p*<.001), physical health (*r* = .53, *p*<.001), psychological condition (*r* = .54, *p*<.001), social (*r* = .37, *p*<.001), and environmental domain (*r* = .56, *p*<.001).

Self-esteem significantly [moderately] correlates with social support (*r* = .63, *p*<.001), quality of life (*r* = .64, *p*<.001), physical health (*r* = .51, *p*<.001), psychological condition (*r* = .56, *p*<.001), social (*r* = .40, *p*<.001), and environmental domain (*r* =.59, *p*<.001).

Social support significantly [moderately] correlates with quality of life (*r* = .74, *p*<.001), physical health (*r* = .58, *p*<.001), psychological condition (*r* = .56, *p*<.001), social (*r* = .55, *p*<.001), environmental features (*r* =.69, *p*<.001).

| **Table 3** | | | | | | | | | |
| --- | --- | --- | --- | --- | --- | --- | --- | --- | --- |
| *Correlations among Demographic and Study Variables of Elderly in Pakistan* (*n* = 557) | | | | | | | | | |
| Variables | QoL | Physical | Psych | Social | Env. | Lifestyle | Efficacy | Esteem | SS |
| Living place | .01 | -.05 | .02 | -.02 | .05 | -.03 | .02 | .03 | .01 |
| Gender | .27** | .25** | .27** | .12** | .19** | .32** | .23** | .17** | -.02 |
| Income | .18** | .14** | .12** | .14** | .16** | .16** | .06 | .10* | .01 |
| Family System | -.15** | -.16** | -.13** | -.14** | -.07 | -.03 | -.15** | -.11* | .03 |
| Children with ASD | .14** | .13** | .14** | .06 | .11** | .06 | .08 | .04 | -.02 |
| Chronic illness | -.26** | -.33** | -.21** | -.10** | -.14** | -.14** | -.14** | -.12** | -.06 |
| *Note*. QoL = quality of life. Psych = psychological, Env. = environmental, Lifestyle = health and lifestyle SS = social support,  ***p*< .01, **p*< .05 (two-tailed). | | | | | | | | | |

Table 3 shows that overall quality of life significantly [low] correlates with gender (*r* = .27, *p*< .01), income (*r* = .18, *p*< .01), family system (*r* = -.15, *p*< .01), children with ASD(*r* = .14, *p*< .01) and health status (*r* = .26, *p*< .01). Physical condition significantly correlates with gender (*r* = .25, *p*< .01), income (*r* = .14, *p*< .01), family system(*r* = -.16, *p*< .01), children with ASD (*r* = .13, *p*< .01) and health status (*r* = .33, *p*< .01). Psychological health significantly correlates with gender (*r* = .27, *p*< .01), income (*r* = .12, *p*< .01), family system(*r* = -.13, *p*< .01), children with ASD (*r* = .14, *p*< .01) and health status (*r* = .21, *p*< .01). Social relations significantly correlate with gender (*r* = .12, *p*< .01), income (*r* = .14, *p*< .01), family(*r* = -.14, *p*< .01), and health status (*r* = .10, *p*< .01). Lastly, environmental features significantly correlates with gender (*r* = .19, *p*< .01), income (*r* = .16, *p*< .01), children with ASD (*r* = .11, *p*< .01) and health status (*r* = .14, *p*< .01).

Health and Lifestyle significantly [low] correlates with gender (*r* = .32, *p*< .01), income (*r* = .16, *p*< .01), and health status (*r* = .14, *p*< .01). Self-Efficacy significantly correlates with gender (*r* = .23, *p*< .01), family system (*r* = -.15, *p*< .01), and health status (*r* = .14, *p*< .01). Self-esteem significantly correlates with gender (*r* = .17, *p*< .01), income (*r* = .10, *p*< .05), family system (*r* = -.11, *p*< .05) and health status (*r* = .12, *p*< .01).

| **Table 4** | | | | | | | | | |
| --- | --- | --- | --- | --- | --- | --- | --- | --- | --- |
| *Correlations among Demographic and Study Variables of Elderly in Canada* (*n* = 448) | | | | | | | | | |
| Variables | QoL | Physical | Psych | Social | Env. | Lifestyle | Efficacy | Esteem | PSS |
| Gender | -.00 | -.00 | .05 | -.01 | -.03 | .00 | .03 | -.04 | -.01 |
| Living place | -.04 | -.01 | -.08 | -.06 | -.01 | .04 | -.07 | -.08 | -.01 |
| Family system | .20** | .20** | .12* | .08 | .20** | .13** | .11* | .24** | .23** |
| Income | .21** | .16** | .18** | .13** | .20** | .15** | .17** | .21** | .13** |
| Children with ASD | .04 | .01 | .09* | -.01 | .02 | .04 | .04 | .05 | .02 |
| Chronic illness | .13** | .09 | .22** | .08 | .07 | .08 | .28** | .08 | .10** |
| *Note*. QoL = quality of life, Psych = psychological, Env.= environmental, Lifestyle = health and lifestyle, SS = social support.  ***p*< .01, **p*< .05 (two-tailed). | | | | | | | | | |

Table 4 shows that overall quality of life significantly [low] correlates with family system (*r* = .20, *p*< .01), income (*r* = .21, *p*< .01), and chronic illness (*r* = .13, *p*< .01). Physical condition significantly correlates with family system (*r* = .20, *p*< .01), and income (*r* = .16, *p*< .01). Psychological health significantly correlates with family system (*r* = .12, *p*< .01), income (*r* = .18, *p*< .01), children with ASD (*r* = .09, *p*< .05) and chronic illness (*r* = .22, *p*< .01). Social relations significantly correlate with family system (*r* = .13, *p*< .01), and income (*r* = .12, *p*< .01). Lastly, environmental features significantly correlate with family system (*r* = .20, *p*< .01), and income (*r* = .20, *p*< .01).

Health and lifestyle significantly [low] correlates with family system (*r* = .13, *p*< .01), and income (*r* = .15, *p*< .01). Self-efficacy significantly correlates with family system (*r* = .11, *p*< .05), income (*r* = .17, *p*< .01), and chronic illness(*r* = .28, *p*< .01). Self-esteem significantly correlates with family system (*r* = .24, *p*< .01) and income (*r* = .21, *p*< .01). Social support significantly correlates with family system (*r* = .23, *p*< .01), income (*r* = .13, *p*< .01) and chronic illness (*r* = .10, *p* < .01).
